# Gene Methylation Affects Salivary Levels of the Taste Buds’ Trophic Factor, Gustin Protein

**DOI:** 10.3390/nu16091304

**Published:** 2024-04-26

**Authors:** Melania Melis, Eleonora Loi, Mariano Mastinu, Lala Chaimae Naciri, Patrizia Zavattari, Iole Tomassini Barbarossa

**Affiliations:** 1 Department of Biomedical Sciences, University of Cagliari, 09042 Monserrato, Italy; eleonora.loi@unica.it (E.L.); l.naciri@studenti.unica.it (L.C.N.); pzavattari@unica.it (P.Z.); tomassin@unica.it (I.T.B.); 2 Smell & Taste Clinic, Department of Otorhinolaryngology, Technical University of Dresden, 01307 Dresden, Germany; mariano.mastinu@ukdd.de

**Keywords:** polymorphism *rs2274333* (A/G) of Gustin (CA6) gene, Gustin gene methylation, salivary Gustin protein expression

## Abstract

The salivary protein, Gustin/carbonic anhydrase VI, has been described as a trophic factor responsible for the growth of taste buds. We found, in a genetically homogeneous population, that the polymorphism *rs2274333* (A/G) of the Gustin gene is crucial for the full functionality of the protein and is associated with taste sensitivity. However, other studies have failed to find this evidence. Here, we verified if Gustin gene methylation can affect the salivary levels of the protein, also concerning the polymorphism *rs2274333* and PROP bitter responsiveness. The Gustin gene methylation profiling and the quantification of the Gustin salivary levels were determined in sixty-six volunteers genotyped for the polymorphism *rs2274333* (A/G) (Ser90Gly in the protein sequence). The fungiform papillae density was also determined. The results confirm our earlier observations by showing that AA genotypes had a greater density of fungiform taste papillae, whereas the GG genotypes showed a lower density. We also found variations in the protein levels in the three genotype groups and an inverse relationship between Gustin gene methylation and the salivary levels of the protein, mostly evident in AA and ST volunteers, i.e., in volunteers who would be carriers of the functional isoform of the protein. These findings could justify the conflicting data in the literature.

## 1. Introduction

Taste is the sensory modality considered to be one of the most relevant factors that influence nutrition and health [1,2,3]. This role is supported by evidence demonstrating how individual differences in taste have a significant impact on food preferences and, consequently, eating behavior. Taste sensitivity can also be impacted by a wide range of diseases and medical procedures [4,5,6,7,8,9]. A decline in taste affects aging because of its strong association with nutritional, metabolic, and cognitive compensatory strategies and its impact on life quality could be substantial [10,11]. Based on this evidence, numerous research endeavors have centered on pinpointing and comprehending the factors that contribute to these significant inter-individual taste variations [12,13,14,15].

For more than 40 years, the zinc-dependent salivary protein, Gustin/carbonic anhydrase VI, has been described as a trophic factor responsible for the growth of taste buds based on observations of patients with taste loss who exhibited pathological changes in their taste buds and low salivary Gustin and zinc levels [16,17]. In our laboratory, the role of Gustin has been studied in a genetically homogeneous population [15,18,19]. The results showed that the presence of the G allele at the *rs2274333* polymorphism of the Gustin gene, leading to a glycine instead of a serine (A allele), potentially results in a disordered/unstructured secondary structure of the protein’s active site [15]. In addition, volunteers carrying the allele A, in homozygosity or heterozygosity, exhibited higher responsiveness to 6-n-propylthiouracil, PROP (PROP super-tasters, STs, and medium tasters, MTs), while those who had the GG genotype showed lower responsiveness (PROP non-tasters, NTs) [15,18]. This suggests that the polymorphism *rs2274333* (A/G) of the Gustin gene led to a modification of the secondary structure of the Gustin protein (Ser90Gly) which is crucial for zinc binding at the active site and protein’s enzymatic activity, which is associated with taste sensitivity. The effect of this polymorphism of the Gustin gene has been also studied on the fungiform papillae density and morphology and in vitro *experiments* on cell proliferation and metabolic activity [19]. The results suggested that the polymorphism affects the density and maintenance of the fungiform papillae and that donors of homozygous AA display an isoform of the protein with full functional activity, in contrast to donors of homozygous GG. These findings offer both physiological and molecular proof of a strong correlation between Gustin gene variants and the development and function of fungiform papillae. However, other studies have failed to find evidence to support significant differences in the fungiform papillae density or taste function associated with Gustin gene polymorphism [20,21].

Environmental exposures may influence gene expression profiles. DNA methylation (DNAm) is an essential epigenetic mark, strongly associated with the regulation of gene expression [22,23]. CpG islands (regions rich in CpG sites of about 200–1000 bp) are usually unmethylated in the promoters of genes actively transcribed. The methylation alteration of several genes has been implicated in various pathologies [24,25,26,27,28]. In addition, genome-wide DNAm changes are linked to aging, quality of life of elderly individuals, and age-related diseases. Therefore, DNAm alterations represent useful biomarkers of biological aging and early disease risk, life expectancy, and mortality assessments [29]. Interestingly, epigenetic changes in two genes encoding lingual lipid sensors (CD36 and GPR120) were associated with the low orosensory perception of fatty acid and a bitter taste [30]. TAS1R2 methylation was correlated with the total energy and carbohydrate dietary intakes [31]. However, no study was conducted on the influence of methylation on taste dysgeusia, representing a potential opportunity for further investigation.

This study aimed to verify whether variations in Gustin expression levels, potentially influenced by different methylation patterns, could justify the discordant data on the effect of the polymorphism *rs2274333* (A/G) of the Gustin gene (Ser90Gly in the protein sequence) on fungiform papillae density and taste function. To achieve this aim, we have analyzed the Gustin gene methylation profiling and salivary levels of the Gustin protein, also considering the polymorphism *rs2274333* and PROP responsiveness. The fungiform papillae density was also determined.

**Figure 1:**
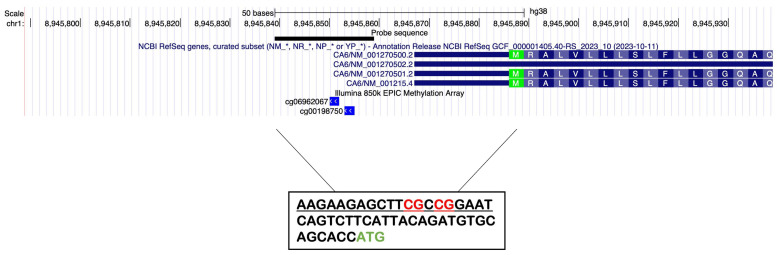
The two CpG sites (cg06962067 and cg00198750) interrogated by MethyLight assay in the region (chr1:8945775-8945902, hg38) upstream of the coding sequence (CDS) of the Gustin gene are located 36 bp and 33 bp upstream of the ATG. The figure is modified from UCSC genome browser.

It is important to note the absence of CpG islands in the Gustin promoter, regions whose association between their level of methylation and gene transcription regulation is well established. However, focusing on the promoter region, there are some scattered CpG loci which could be involved in the regulation of gene expression. Therefore, the first purpose of this work was to interrogate the DNAm status of two CpG sites located in the region upstream of the coding sequence (CDS) of the Gustin gene with a specific assay. We have chosen to focus our analysis on the region surrounding the transcription start site (TSS). Indeed, the two selected CpG loci are located 36 bp and 33 bp upstream of the start of CDS, ATG (Figure 1). They are also the closest ones to the CDS among the CpGs interrogated by Illumina EPIC array probes, reinforcing the robustness of the assay design and the usability of the methylation data produced in our work. In addition, it has been well demonstrated that the methylation level of the region upstream of the CSD highly influences gene expression [32] and that it is much more tightly associated with gene expression silencing than methylation in the promoter region [33,34].

## 2. Materials and Methods

### 2.1. Volunteers and Experimental Procedure

Sixty-six Caucasian volunteers (48 females and 18 males, ages 24.76 ± 4.80) were recruited at the University of Cagliari using standard procedures. They were originally from Sardinia, Italy. Volunteers were normal weight with a body mass index (BMI) ranging from 20.2 to 24.8 kg/m^2^. Exclusion criteria included pregnancy, lactation, major metabolic diseases (diabetes, kidney disease, etc.), food allergies, and the usage of drugs that would have impaired their ability to taste or smell (e.g., steroids, antihistamines, or anti-depressants). Their taste function for the four basic tastes was evaluated using the taste strip test (Burghart Messtechnik, Wedel, Germany) to rule out any taste impairment. All volunteers were informed regarding the purpose and procedure of the study. All volunteers provided written informed consent. The current study was carried out following the most recent version of the Helsinki Declaration, and all methods were authorized by the University Hospital Company’s (AOU) Ethical Committee in Cagliari, Italy (protocol code 451/09, date of approval May 2016).

All volunteers were instructed to abstain from eating, drinking (except water), using dental care products, or chewing gum for at least two hours before testing. Each volunteer was required to arrive in the experiment room fifteen minutes before the test’s scheduled start time (9.30 a.m.) to adapt to the environmental conditions (23–24 °C; 40–50% relative humidity and light with standard solar light 15,000 lux) that were kept constant throughout the experimental session. The environment was maintained relatively quiet and odor-free. For each volunteer, multiple photographs of the tongue surface were taken to determine the density of fungiform papillae. All were assessed for their PROP responsiveness and two samples of the whole-unstimulated saliva (1 mL each) were collected as described below. To prevent alterations in taste function caused by the estrogenic phase, women were tested around the sixth day of the menstrual cycle [35].

### 2.2. PROP Responsiveness Measurements

PROP responsiveness of each volunteer was assessed by a scaling method for their taster status classification. The impregnated paper screening test [36], which had been tested for validity and reliability [37,38,39], was used. The test is based on the ratings of two paper disks, placed for 30 s on the tip of the tongue, impregnated with sodium chloride, NaCl (1.0 mol/L), and PROP solution (50 mmol/L), respectively. Intensity ratings for PROP or NaCl were collected using the Labeled Magnitude Scale (LMS) [40]. Volunteers were trained in the use of the LMS before testing. This scale gives volunteers the freedom to rate the perceived taste intensity for each stimulus relative to the “strongest imaginable” oral stimulus they had ever experienced in their lives. The interstimulus interval was set at 5 min. Volunteers who evaluated the PROP disk higher than 67 mm in LMS were classified as PROP super-tasters (STs), those who evaluated the PROP lower than 15 mm on the scale were classified as PROP non-tasters (NTs), and those who rated the PROP disk with intermediate ratings were classified as medium-tasters (MTs) [36]. Volunteers who rated the PROP disk as borderline (≅15 mm) and rated NaCl significantly higher (≅30 mm discrepancy on the LMS) were classified as NT. Likewise, volunteers who rated the PROP disk as borderline (≅67 mm) and rated NaCl much lower were classified as ST.

Based on their taster group assignments, 27 volunteers were classified as ST (40.91%), 29 were MT (43.94%), and 10 were NT (15.15%).

### 2.3. Density Assessments of the Fungiform Taste Papillae

Fungiform papillae density was determined according to Melis et al. 2013 [19] on the anterior tongue surface’s tip, at the left side of the midline. This area provides measurements of the fungiform papillae density which are highly correlated with the total number on the tongue [41]. The tip of the tongue was dried with filter paper and stained by placing (for 3 s) a piece of filter paper (circle 6 mm in dia) that was impregnated with blue food coloring (E133, Modecor Italiana, Cuvio, Italy). Multiple photographs of the stained area were taken using a Nikon Coolpix P520 Digital Camera (Nikon Corporation, Konan, Minato-ku, Tokyo) (18.1 megapixels). After downloading the digital photos to a computer, the “zoom” feature of Adobe Photoshop CS2 version 9.0 (Adobe Systems Incorporated, San Jose, CA, USA) was used to evaluate the pictures. The fungiform papillae in the stained area were identified by their mushroom shape and distinguished by their very light staining in contrast to filiform papillae which stained dark [42]. The number of papillae was counted for each volunteer separately by three trained observers who were blind to the genotype of SNP analyzed and to the PROP taster status of volunteers [19,39,41]. Final measurements were based on the consensus of all observers which then calculated the density/cm^2^.

### 2.4. Salivary Levels of Gustin Protein

#### 2.4.1. Saliva Collection and Treatment

Using a soft plastic aspirator, two samples (1 mL) of whole unstimulated saliva were collected, within less than a minute, from the anterior floor of the mouth of each volunteer. One tube was directly stored at −80 °C until the DNA extraction, and the second was immediately treated for the immunoblot procedure for Gustin protein quantification before storage. A protease inhibitor cocktail solution [mix of 1 tablet/1.4 mL of cOmplete^®^ Protease Inhibitor Cocktail (Roche Diagnostics, Indianapolis, IN, USA) and 175 mM NH_4_HCO_3_ (Ammonium Bicarbonate, Sigma-Aldrich, St. Louis, MO, USA) was added to saliva in a 1:2 *v/v* ratio.

#### 2.4.2. Salivary Protein Quantification

Bicinchoninic Acid (BCA) Protein Assay Kit (Sigma-Aldrich, St. Louis, MO, USA) was used to quantify the total protein content of the saliva samples according to the manufacturer’s instructions. The total concentration of salivary protein was used to normalize the protein levels in the dot blot analysis.

The quantification of the Gustin protein salivary levels was performed using the semi-quantitative dot-blot technique where the protein samples were spotted directly onto a PVDF membrane (0.2 μm pore size; Immun-Blot^®^ PVDF Membrane, Bio-Rad Laboratories, Inc., Segrate, Italy). To prepare an assay for dot blot, the treated saliva samples were first diluted with Tris Buffered Saline (TBS: 20 mM Tris-HCl pH 7.6, 150 mM NaCl) to ensure that every sample had the same amount of total protein content (adjusted to 0.42 μg/μL). The recombinant Human Carbonic Anhydrase VI Protein (code: 2939-CA-010, R&D Systems, Minneapolis, MN, USA) was used as a standard in 5 concentrations (0.2, 0.3, 0.4, 0.6, and 0.8 µg/µL).

Before transferring the samples onto the PVDF membrane, it was pre-wetted with methanol for 1 min, and then transferred to TBS for 2 min. All samples (2 μL of volume) and standards (1 μL of volume) were spotted onto the wet PVDF membrane in triplicate. The membrane was blocked with a blocking agent of 5% of BSA (Bovine serum albumin, Sigma Aldrich, St. Louis, MO, USA) in TBS-T buffer (20 mM Tris-HCl pH 7.6, 150 mM NaCl, 0.05% Tween 20) for 1 h at room temperature. Subsequently, the membrane was incubated for 2 h at room temperature with primary antibody (0.5 µg/mL, Human Carbonic Anhydrase VI antibody, Monoclonal Mouse IgG3 clone #401819, Catalog: MAB2939 R&D Systems) in 0.5% of BSA in TBS-T buffer. Three washes for 5 min with TBS-T buffer were performed and the membrane was incubated for 1 h with secondary antibody (dilution 1:5000; Rabbit anti-Mouse IgG, Secondary Antibody, HRP Thermo Fisher Scientific, Waltham, MA, USA). After three further washes with TBS-T and one wash with TBS (5 min), the membrane was incubated for 5 min with ECL substrate (Clarity Western ECL Substrate, Bio-Rad Laboratories, Inc., Segrate, Italy) to develop fluorescence signals and captured on the iBright™ CL1500 Imaging System (ThermoFisher Scientific, Waltham, MA, USA). Analysis of images obtained was performed using iBright Analysis Software Desktop Version 5.2.0 (ThermoFisher Scientific, Waltham, MA, USA). Signals of samples were identified and shown as intensity values which were transformed by the software into value of concentration (μg/μL) for each sample by using the standards as references. Each sample was analyzed three times, with a reasonable coefficient of variation (CV%) set as below 15%.

### 2.5. Genotyping for Gustin (CA6) Gene Polymorphism rs2274333

DNA was extracted from saliva samples using the standard salting-out procedure. The concentration of purified DNA was estimated by measuring the optical density at 260 nm with a NanoDrop™ One/OneC Microvolume UV-Vis Spectrophotometer (Thermo Scientific™, Life-Technologies Italia, Milan, Italy, Europe BV, Monza, Italy). Volunteers were genotyped for the single-nucleotide polymorphism (SNP) *rs2274333* (A/G) of the Gustin gene located in the exon 3 that resulted in a substitution of amino acid Ser90Gly in the protein sequence by using TaqMan^®^ SNP Genotyping Assay technique, using the assay with code: C_1739329_1 (ThermoFisher Scientific, Waltham, MA, USA). The plates were read using a StepOne™ Real-Time PCR System following the manufacturer’s instructions (Applied Biosystems by Life Technologies, Milan, Italy, Europe BV, Monza, Italy). The results were analyzed by allelic discrimination of the sequence detector software (TaqMan^®^ Genotyper Software v1.7.1, Applied Biosystems, Inc., Foster City, CA, USA). Replicates, two negative and three positive controls (one for each genotype), were included in all reactions. The PCR reactions were run on 96-well plates with fast thermal cycling conditions and included: 1X TaqMan^®^ genotyping master mix (code: 4371355), 1X TaqMan^®^ genotyping assays (C_1739329_1, Catalog number: 4351379), and 10 ng of DNA and nuclease-free water.

In total, 39 (59 %) participants had the AA genotype (16 were classified as STs, 19 MTs, and 4 NTs), 21 (32 %) had the AG genotype (10 STs, 8 MTs, and 3 NTs), and 6 (9 %) had the GG genotype (1 ST, 2 MTs, and 3 NTs). Although Allele A was more frequent in STs and MTs (78% and 79%) than in NTs (55%), no significant differences were found based on the genotype distribution or allelic frequency of the Gustin (CA6) gene polymorphism *rs2274333* (A/G) (Genepop software version 4.2; online software: http://genepop.curtin.edu.au/genepop_op3.html (accessed on 23 February 2024); Montpellier, France).

### 2.6. Gustin Gene Methylation Profiling

Focusing on the promoter region, there are some scattered CpG loci which could be involved in the regulation of gene expression. In particular, in an interval of 1000 bp upstream of the beginning of the coding sequence there are eight CpG loci distant from each other, except the two closest to the ATG (33 and 36 bp upstream of it), and very close to each other (3 bp). This allowed us to design an assay for MethyLight that interrogated at least two CpG loci, as is very often done even when designing an assay within a CpG island. We designed a MethyLight assay (amplicon size 128 bp) to interrogate the DNA methylation status of two CpG sites (cg06962067 and cg00198750) located in the region (chr1:8945775-8945902, hg38) upstream of the coding sequence (CDS) of the Gustin gene (Table 1). The two selected CpG loci are located 36 bp and 33 bp upstream of the ATG. The designed assay involves the use of primers that hybridize to regions not influenced by methylation (not containing CpG loci) and a probe that hybridizes in the stretch of sequence containing the two CpG loci described above, capable of discriminating the methylated from the unmethylated state.

We carried out two reactions for each sample: one for the target assay and one for the bisulfite-dependent methylation-independent control (ALU-C4) that was used to normalize the quantity of the input DNA sample [43]. Each reaction was performed in triplicate and contained: 1X TaqMan^®^ Genotyping Master mix (Applied Biosystems, Foster City, CA, USA), 900 nM of each primer, 250 nM of probe, and 30 ng bisulfite-converted DNA in a final volume of 20 μL. The experiment was conducted on Rotor-Gene Q (Qiagen, Venlo, The Netherlands) using the following thermal conditions: 95 °C for 10 min, followed by 45 cycles of 95 °C for 15 sec and 60 °C for 1 min. The methylation levels were expressed as Δ cycle threshold (Ct), calculated as the difference between Ct of the target assay and Ct of the ALU-C4 control (higher ΔCt means a lower methylation level and vice versa).

### 2.7. Statistical Analyses

One-way ANOVA was used to analyze the effect of Gustin gene polymorphism *rs2274333* on the density of fungiform papillae and salivary levels of Gustin protein and to compare the differences in salivary levels of Gustin protein among ST, MT, and NT volunteers. Post hoc comparisons were conducted with Fisher’s least significant difference (LSD) test. The relationships between salivary levels of Gustin protein and ∆Ct values were assessed using Pearson linear correlation analysis. The same analysis was conducted separately in the three Gustin genotypes and PROP taster groups.

Statistical analyses were conducted using STATISTICA for WINDOWS (version 7; StatSoft Inc., Tulsa, OK, USA). *p* values ≤ 0.05 were considered significant.

## 3. Results

**Figure 2:**
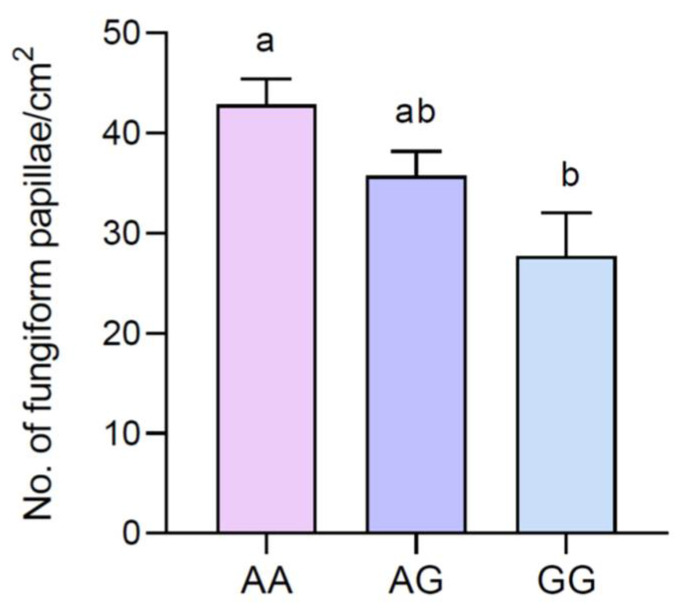
Mean values ± SEM of the density of fungiform papillae (No./cm^2^) of volunteers with AA, AG, and GG genotypes of Gustin polymorphism *rs2274333* (A/G). *n* = 66. Different letters indicate a significant difference (*p* = 0.019; Fisher LSD test after one-way ANOVA).

The mean values of the densities (±SEM) of the fungiform papillae of participants with genotypes AA, AG, and GG of the Gustin gene are shown in Figure 2. One-way ANOVA revealed that the fungiform papillae density was associated with Gustin gene polymorphism (F_(2,63)_ = 3.788; *p* = 0.029). Specifically, the values of the fungiform papillae density were lower in volunteers with the GG genotype than in those with the AA genotype (*p* = 0.019; Fisher LSD test). The values of the AG genotypes were not different from those of the other groups (*p* > 0.05).

**Figure 3:**
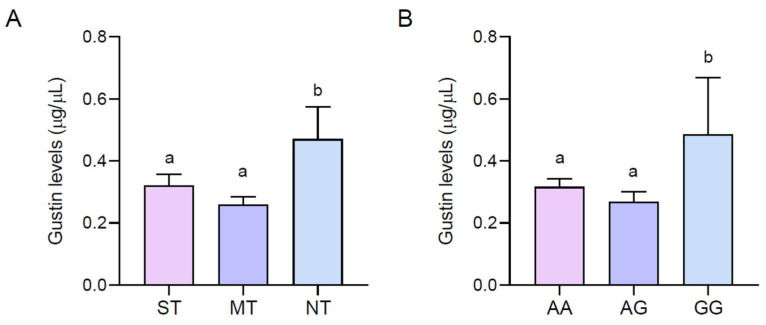
Mean values ± SEM of salivary levels of Gustin protein (µg/µL) for STs, MTs, and NTs (**A**) and for volunteers with genotypes AA, AG, and GG (**B**) of Gustin polymorphism *rs2274333* (A/G) *n* = 66. Different letters indicate a significant difference (*p* ≤ 0.037; Fisher LSD test after one-way ANOVA).

Figure 3 shows the mean values of the salivary levels of the Gustin protein (µg/µL) of PROP ST, MT, and NT (A) volunteers and with the AA, AG, and GG genotypes of the Gustin gene (B). ANOVA revealed that the salivary levels of the Gustin protein were associated with PROP responsiveness (F_(2,63)_ = 4.408; *p* = 0.016). A post hoc comparison showed that the salivary levels of the Gustin protein were higher in NT volunteers than those in MT and ST volunteers (*p* ≤ 0.042, Fisher LSD test). A post hoc comparison also showed that the salivary levels of the Gustin protein were higher in volunteers with the GG genotype than in those with the AA or AG genotypes (*p* ≤ 0.037, Fisher LSD test), but there was no difference between the AA and AG volunteers (*p* > 0.05).

**Figure 4:**
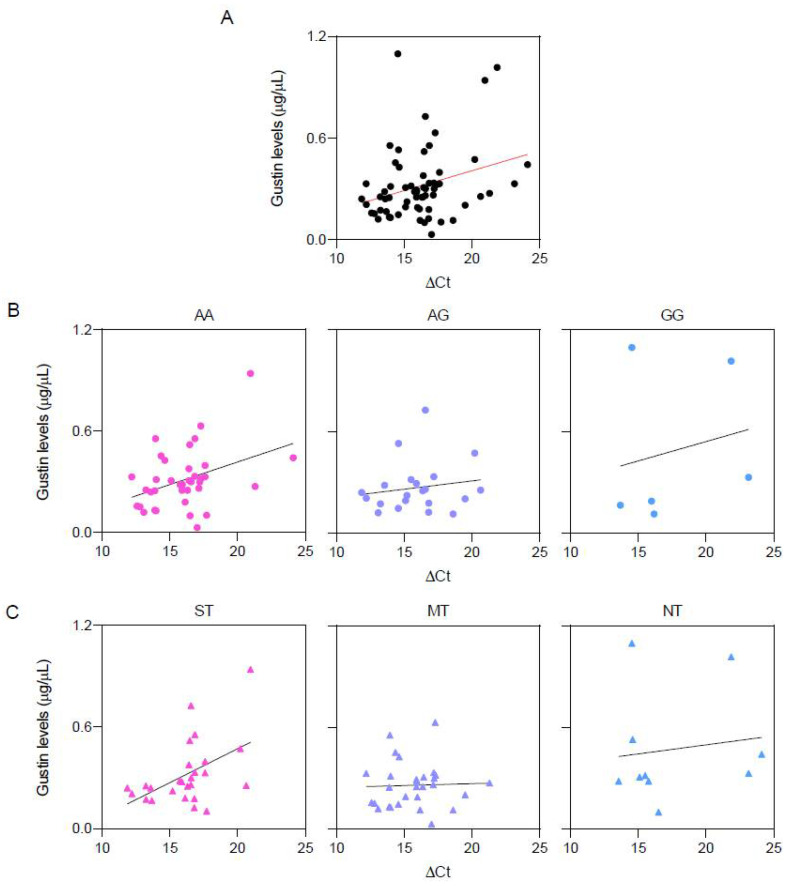
Pearson linear correlation analysis between the salivary levels of Gustin protein (µg/µL) and ∆Ct values. (**A**) Relationship in the whole sample (r = 0.304; *p* = 0.015). (**B**) Relationships in the three Gustin genotype groups (genotype AA: r = 0.380; *p* = 0.018; genotype AG: r = 0.152; *p* > 0.05; genotype GG: r = 0.201; *p* > 0.05). (**C**) Relationships in the three PROP taster groups (ST: r = 0.490; *p* = 0.011; MT: r = 0.056; *p* > 0.05; NT: r = 0.127; *p* > 0.05). *n* = 66.

Figure 4 shows the scatterplots depicting the relationships between the salivary levels of the Gustin protein (µg/µL) and ∆Ct values in the whole sample (A), in the three Gustin genotype groups (B), and the PROP taster groups (C). A linear correlation analysis showed that the salivary levels of the Gustin protein (µg/µL) were linearly correlated with the ∆Ct values in the whole sample (r = 0.304; *p* = 0.015), in volunteers with the AA genotype (r = 0.380; *p* = 0.018), and PROP ST (r = 0.490; *p* = 0.011). No correlation was found between the salivary levels of the Gustin protein and the ∆Ct values in the AG and GG groups or MT and NT volunteers (*p* > 0.05).

## 4. Discussion

The primary aim of the present work was to verify whether variations in Gustin gene expression, which could be influenced by different methylation patterns, could represent a confounding factor for the effect of the polymorphism *rs2274333* (A/G) gene (Ser90Gly in the protein sequence) on the fungiform papillae density and taste function, which may explain discrepant findings across the population.

First of all, it has been fundamental to select the two CpG sites (cg06962067 and cg00198750), located in the region upstream of the coding sequence (CDS) of the Gustin gene, whose methylation status could potentially affect gene expression. We found a direct correlation between the ∆Ct values and the salivary levels of the Gustin protein in the whole sample, indicating higher methylation associated with a lower amount of protein. Therefore, this result strongly indicates a potential involvement of these two CpG sites in regulating gene expression. This is not surprising since it is generally known that methylation in the region upstream of the CSD significantly influences gene expression [32] and is more strictly linked to the silencing of gene expression than methylation in the promoter region [33,34].

In our previous works, we studied the effect of the *rs2274333* (A/G) polymorphism in the Gustin gene (Ser90Gly in the protein sequence) on PROP responsiveness and its role as a trophic factor for cell development and the maintenance of fungiform papillae in a genetically homogeneous cohort. First, Padiglia et al. 2010 [15] revealed that the GG genotype was associated with structural alterations and the decreased functionality of the protein, and PROP NTs were more likely to possess this genotype. Afterward, Calò et al. 2011 [18] showed that having two A alleles increased the PROP bitterness intensity regardless of the genotype groups of the specific receptor TAS2R38. Finally, Melis et al. 2013 [19], in in vivo and in vitro experiments, showed that the polymorphism of the Gustin gene influences PROP responsiveness by acting on the fungiform papillae density and maintenance, and the treatment of isolated cells with the active isoform of the Gustin protein increased cell division and metabolic activity. Otherwise, other authors failed to find associations between Gustin polymorphism and PROP responsiveness or papillae density in ethnically mixed populations in Brazil [20] and in the U.S. [21]. Specifically, Feeney and Hayes 2014 [21] found no difference in PROP bitter taste perception and fungiform papillae density for the Gustin genotypes. Also, they failed to find any evidence that Gustin’s effects on taste perception can be due to differences in the fungiform papillae density. Genick et al. 2011 [20], in a genome-wide study, showed no associations between Gustin and PROP phenotypes.

The results of the present work confirm our earlier observations by showing that volunteers carrying the AA genotype, who should express the functional form of the Gustin protein, had a greater density of fungiform taste papillae, whereas those carrying the GG genotype, who should express a less functional isoform, showed a lower density. We also found variations in protein levels in the three genotype groups which can limit the effect of the genotype on papillae density. Gustin protein levels were higher in volunteers carrying the GG genotype (who have the less functional isoform of protein and show a lower density) compared with volunteers carrying the AA or AG genotypes (who have the functional isoform of protein and show a higher density). Interestingly, Gustin protein salivary levels were higher in volunteers who showed low PROP responsiveness (NT) than in volunteers who showed higher PROP responsiveness (MT and ST).

In addition, our results showed an inverse relationship between Gustin gene methylation and the salivary levels of the Gustin protein, indicating higher methylation (and thus usually the lower expression of the gene) associated with a lower amount of protein. We found high ∆Ct values associated with the high salivary levels of the Gustin protein and low ∆Ct values associated with low protein levels (a higher ΔCt means a lower methylation level and vice versa). It is worth noting that this was particularly evident in AA volunteers who are carriers of the functional isoform of the Gustin protein. This suggests that the levels of the DNAm, and thus of the salivary protein, are important in contributing to the effect of Gustin as a trophic factor in those volunteers who are carriers of the functional form of the protein. On the other hand, the levels of DNAm seem to be insignificant in volunteers carrying the non-functional form (AG and GG volunteers). Therefore, also when the levels of methylation are low, determining a high amount of the protein (GG volunteers), its effect as a trophic factor is not observable because it is in its nonfunctional form. These findings should be confirmed in a larger population and in ethnically mixed populations to verify this hypothesis. The effect of DNAm was particularly evident also in ST volunteers who showed the highest PROP responsiveness. Since PROP tasting is considered a paradigm of general taste function and an oral marker for food preferences [1], this result suggests that the methylation of the Gustin gene, and thus its expression, is important for the volunteers who have a more developed gustatory function.

## 5. Conclusions

In conclusion, our findings showed, for the first time, significant correlations between Gustin gene methylation patterns and the effect of the two isoforms of the Gustin protein on the fungiform papillae density and taste function. This epigenetic basis for the effect of the Gustin protein, perhaps responsible for some of the differences among individuals, could help, at least partially, in explaining some of the discrepancies among studies.

## Figures and Tables

**Table 1:** Gustin methylation assay.

Type	Seq (5′→3′)	Tm (°C)
Primer Forward	AAGGGTGGTTTAGTTAGTTGGGTAG	57.1
Primer Reverse	ACACCAAAACCCTCATAATACTACACA	58.0
Probe	5′6-FAM AAGAAGAGTTTCGTCGGAAT	68.0

## Data Availability

The data presented in this study are available on request from the corresponding author. The data are not publicly available, in accordance with consent provided by participants on the use of confidential data.
